# Illegitimate tasks and work role performance among engineering consulting professionals: integrating work–family boundary and cognitive–affective personality system theories

**DOI:** 10.3389/fpsyg.2026.1924102

**Published:** 2026-08-18

**Authors:** Tao Yang

**Affiliations:** Guangxi Logistics Vocational and Technical College, Guigang, Guangxi, China

**Keywords:** authoritarian leadership, cognitive–affective personality system theory, illegitimate tasks, work engagement, work role performance, work–family boundary theory, work–life balance

## Abstract

**Introduction:**

Professionals are often required to perform tasks they regard as unnecessary or inconsistent with their legitimate responsibilities. Although research has linked such illegitimate tasks to adverse work outcomes, less is known about how they are associated with employees’ appraisal of the work–nonwork interface, motivational investment, and work role performance, or how leadership conditions this process. Drawing primarily on cognitive–affective personality system (CAPS) theory, we propose that illegitimate tasks operate as situational stimuli that activate employees’ work–life balance appraisals, which are associated with work engagement and, in turn, employee-reported work role performance. Work–family boundary theory is used to explain why authoritarian leadership strengthens the negative association between illegitimate tasks and work–life balance.

**Methods:**

Using three-wave survey data from 615 engineering consulting professionals, we tested a moderated, theoretically ordered indirect-effects model examining the associations among illegitimate tasks, work–life balance, work engagement, employee-reported work role performance, and authoritarian leadership.

**Results:**

Illegitimate tasks were negatively associated with work–life balance; work–life balance was positively associated with work engagement; and work engagement was positively associated with employee-reported work role performance. The serial indirect association through work–life balance and work engagement was negative and became stronger as authoritarian leadership increased.

**Discussion:**

These findings identify work–life balance as one complementary cross-domain pathway through which illegitimate tasks may be associated with engagement and performance. They also show how CAPS accounts for the proposed stimulus–appraisal–motivation–performance process, whereas work–family boundary theory explains the leadership condition that strengthens its initial association. Because work–life balance and work engagement were assessed during the same survey wave, the findings support a theoretically ordered indirect association rather than a definitive temporal or causal sequence.

## 1 Introduction

Professional service firms depend on employees whose value lies not simply in completing assigned work, but in sustaining concentrated judgment across complex and uncertain tasks (Alvesson and Sveningsson, 2003; Von Nordenflycht, 2010). Yet professionals are routinely pulled away from their core responsibilities by repetitive reporting, procedural coordination, and temporary support work that they may regard as unnecessary or inconsistent with their legitimate roles. Such demands are not innocuous administrative burdens. They interrupt attention and task continuity (Baethge et al., 2016; Beal et al., 2005), and, when perceived as lacking necessity or role legitimacy, constitute illegitimate tasks (Semmer et al., 2015). Their consequences may also extend beyond the immediate work episode. Prior research has associated illegitimate tasks with adverse affective experiences, work attitudes, and work–family outcomes (Ahmed et al., 2018; Eatough et al., 2016; Ma and Peng, 2019), consistent with broader evidence that work demands can spill over into employees’ nonwork lives (Allen et al., 2000; Amstad et al., 2011). This creates a consequential organizational tension: professional service firms rely on sustained attention and judgment while simultaneously imposing low-value or role-inconsistent demands that can displace the very activities on which professional performance depends (Ding and Kuvaas, 2023).

Recent research further shows that employees do not respond uniformly to illegitimate tasks. Chen et al. (2025) linked illegitimate tasks to supervisor-directed deviance through negative reciprocity and identified psychological hardiness as a buffering condition. Cheng et al. (2025) found that illegitimate tasks may either promote or inhibit adaptive performance depending on employees’ general self-efficacy and coping responses. Ma et al. (2026) associated illegitimate tasks with quiet quitting through relative deprivation, with neuroticism strengthening this association, whereas Tian et al. (2026) examined how need for achievement and coping strategies shape employees’ divergent responses to illegitimate tasks. Together, these studies underscore the importance of appraisal, coping, and individual differences in explaining how employees respond to such demands.

These emerging explanations complement earlier evidence that illegitimate tasks elicit adverse affective and cognitive reactions and affront employees’ self-worth, occupational identity, and sense of fairness (Ahmed et al., 2018; Ding and Kuvaas, 2023; Ma and Peng, 2019; Semmer et al., 2010). Yet a related question remains less settled: how are these demands associated with professionals’ capacity to sustain effective role performance when they also complicate the coordination of work and nonwork activities? This question matters because professional performance requires continued psychological investment in core work, and demands that diminish employees’ perceived work–life compatibility may make such investment more difficult to sustain (Crawford et al., 2010). We address this question by examining work–life balance as employees’ appraisal of compatibility between work and nonwork activities and work engagement as the motivational state associated with that appraisal. This cross-domain pathway is intended to complement, rather than replace, existing affective, identity-based, fairness-based, and coping-based explanations of illegitimate tasks.

To explain the proposed indirect process, we draw on cognitive–affective personality system (CAPS) theory as the primary theoretical framework. CAPS proposes that situational features activate interconnected cognitive and affective–motivational units that are associated with behavioral responses (Mischel and Shoda, 1995; Shoda and Mischel, 2000). In the present model, illegitimate tasks constitute the situational stimulus; work–life balance represents employees’ cognitive appraisal of whether their work and nonwork activities remain compatible; work engagement represents the affective–motivational unit reflected in vigor, dedication, and absorption; and employee-reported work role performance represents the behavioral outcome. Accordingly, CAPS explains the full theoretically ordered indirect process through which illegitimate tasks are associated with work role performance via work–life balance and work engagement.

Work–family boundary theory is used for a separate and narrower purpose: to explain why authoritarian leadership strengthens the negative association between illegitimate tasks and work–life balance. From this perspective, authoritarian leaders operate as work-domain boundary managers who restrict employees’ latitude to negotiate task ownership, completion timing, and priorities (Ashforth et al., 2000; Cheng et al., 2004; Clark, 2000). When this latitude is restricted, illegitimate tasks become more difficult to accommodate within employees’ existing work and nonwork arrangements and are therefore more strongly associated with unfavorable work–life balance appraisals. Thus, work–family boundary theory explains the first-stage moderating effect of authoritarian leadership; it is not used to explain the subsequent associations from work–life balance to work engagement and work role performance.

We examine these arguments using three-wave survey data from 615 professionals in the engineering consulting industry. The study tests a theoretically ordered indirect pathway linking illegitimate tasks to employee-reported work role performance through work–life balance and work engagement, together with the moderating role of authoritarian leadership in the first stage of this pathway. In doing so, the study makes three contributions. First, the study establishes a clear division of theoretical labor between CAPS and work–family boundary theory. CAPS serves as the primary theory explaining the full stimulus–appraisal–motivation–performance process: illegitimate tasks operate as situational stimuli, work–life balance represents a cognitive appraisal, work engagement represents an affective–motivational unit, and employee-reported work role performance represents the behavioral outcome. Work–family boundary theory serves only to explain why authoritarian leadership, as a work-domain boundary management condition, strengthens the initial negative association between illegitimate tasks and work–life balance. Second, the study extends illegitimate-task research to the work–nonwork interface by identifying work–life balance as a complementary cross-domain cognitive appraisal pathway through which these tasks may be associated with engagement and performance, complementing rather than displacing established affective, identity-based, and fairness-based explanations. Third, the study identifies authoritarian leadership as a work-domain boundary condition that determines employees’ latitude to negotiate task ownership, completion timing, and priorities. This boundary-management perspective clarifies when illegitimate tasks are more strongly associated with unfavorable work–life balance appraisals.

## 2 Theoretical background and hypotheses

### 2.1 CAPS as the process theory and work–family boundary theory as the moderation theory

CAPS provides the primary theoretical account of the proposed indirect process. The theory holds that situational stimuli activate interconnected cognitive and affective–motivational units, and that these activation patterns are associated with behavioral responses (Mischel and Shoda, 1995; Shoda and Mischel, 2000). In our model, illegitimate tasks constitute the situational stimulus, work–life balance represents the cognitive appraisal, work engagement represents the affective–motivational unit, and employee-reported work role performance represents the behavioral outcome. Work–family boundary theory serves a more focused role by explaining why authoritarian leadership strengthens the initial association between illegitimate tasks and work–life balance (Ashforth et al., 2000; Clark, 2000).

Within CAPS, illegitimate tasks function as situational stimuli because they introduce demands that employees regard as unnecessary or inconsistent with their legitimate responsibilities. Such assignments may require employees to rearrange existing priorities, devote time to low-value activities, or accommodate work they believe should be performed differently or by someone else. Work–life balance captures employees’ overall appraisal of whether their work and nonwork activities remain compatible under these conditions (Brough et al., 2014; Greenhaus et al., 2003). Work–family boundary theory becomes relevant at this point because authoritarian leaders act as work-domain boundary managers who shape employees’ latitude to question, discuss, reschedule, or reorganize assigned work (Ashforth et al., 2000; Cheng et al., 2004; Clark, 2000).

CAPS also explains how the activated work–life balance appraisal is associated with subsequent affective–motivational and behavioral outcomes. The theory holds that situational experiences activate interconnected cognitive and affective–motivational units, and that these activation patterns are associated with behavioral responses (Mischel and Shoda, 1995; Shoda and Mischel, 2000). In this study, work–life balance serves as the cognitive appraisal because it reflects employees’ evaluation of work–nonwork compatibility. Work engagement serves as the affective–motivational state because vigor, dedication, and absorption reflect the psychological energy and involvement employees direct toward their work (Schaufeli et al., 2002). A less favorable appraisal of work–life balance may therefore be associated with lower engagement and, through engagement, lower employee-reported work role performance. This ordering does not imply that cognition invariably precedes affect. Because work–life balance and work engagement were measured during the same survey wave, their ordering remains theoretically specified rather than temporally established.

### 2.2 The theoretically ordered indirect pathway

Consistent with CAPS, illegitimate tasks constitute situational stimuli that activate employees’ appraisal of whether their work and nonwork activities remain compatible (Mischel and Shoda, 1995; Shoda and Mischel, 2000). Work–life balance refers to employees’ subjective appraisal of the extent to which these activities are compatible, rather than to an objective division of time or a direct assessment of boundary control (Brough et al., 2014; Greenhaus et al., 2003). Unnecessary tasks can weaken this appraisal by occupying time and attention that employees might otherwise devote to core work, recovery, or nonwork responsibilities. Unreasonable tasks create a related coordination problem by requiring employees to accommodate demands they regard as inconsistent with their legitimate responsibilities (Semmer et al., 2010). Although the two forms differ in content, both can require employees to rearrange priorities and adjust the coordination of work and nonwork activities. Employees who encounter such demands should therefore report less favorable work–life balance (Ahmed et al., 2018; Eatough et al., 2016; Ma and Peng, 2019).

CAPS explains how employees’ appraisal of work–life balance is associated with their subsequent motivational investment in work. Within CAPS, cognitive appraisals are interconnected with affective–motivational units that organize the energy and involvement employees direct toward a situation (Mischel and Shoda, 1995; Shoda and Mischel, 2000). When employees evaluate their work and nonwork activities as compatible, they may be better positioned to sustain the vigor, dedication, and absorption that characterize work engagement (Schaufeli et al., 2002; Sonnentag and Fritz, 2015). A less favorable work–life balance appraisal, by contrast, may be associated with less psychological energy and attention available for core work.

Work engagement should, in turn, be positively associated with work role performance. Work role performance encompasses proficiency, adaptivity, and proactivity in carrying out one’s role (Griffin et al., 2007). These forms of performance require employees to remain attentive, persistent, and willing to invest effort beyond minimum task completion. Consistent with this reasoning, work engagement has been linked to sustained attention and performance across occupations and performance criteria (Beal et al., 2005; Christian et al., 2011; Corbeanu and Iliescu, 2023; Neuber et al., 2022). Employees who report lower work–life balance should therefore also report lower engagement and, through that association, lower employee-reported work role performance.

Taken together, CAPS organizes the entire proposed indirect pathway: illegitimate tasks function as situational stimuli that activate work–life balance appraisals, which are associated with work engagement and, in turn, employee-reported work role performance. Work–family boundary theory serves specifically to explain why authoritarian leadership conditions the initial stimulus–appraisal association. Because work–life balance and work engagement were measured concurrently, the proposed sequence represents a theoretically specified indirect association rather than an established temporal process.

*Hypothesis 1*: Illegitimate tasks are negatively associated with employee-reported work role performance through a CAPS-based, theoretically ordered indirect pathway involving lower work–life balance and, in turn, lower work engagement.

### 2.3 Authoritarian leadership as a work-domain boundary condition

Work–family boundary theory suggests that employees’ ability to coordinate work and nonwork activities depends partly on whether work demands can be discussed, rescheduled, or reorganized (Ashforth et al., 2000). Authoritarian leaders act as work-domain boundary managers who shape employees’ discretion to negotiate task ownership, completion timing, and priorities. By concentrating authority and emphasizing obedience, authoritarian leaders restrict employees’ latitude to clarify task ownership, negotiate deadlines, and reprioritize competing demands (Cheng et al., 2004). Under less authoritarian leadership, employees may be able to treat an unnecessary or role-inconsistent assignment as temporary or open to adjustment. Under more authoritarian leadership, the same assignment is more likely to be understood as a fixed requirement that must be accepted as given. Authoritarian leadership therefore makes illegitimate tasks less negotiable and more difficult to accommodate within employees’ existing work–life arrangements.

The moderating role of authoritarian leadership should therefore be most evident in the initial association between illegitimate tasks and work–life balance. When authoritarian leadership is low, employees may have greater latitude to clarify task ownership, negotiate completion timing, or rearrange priorities, making illegitimate tasks easier to accommodate alongside existing work and nonwork activities. When authoritarian leadership is high, these options are more restricted because compliance takes precedence over discussion or adjustment (Cheng et al., 2004). As a result, the same illegitimate task is more likely to be associated with an unfavorable appraisal of work–life balance. In boundary-theory terms, authoritarian leadership restricts employees’ ability to coordinate and renegotiate demands across domains. The moderation is therefore located on the first-stage path rather than on the later association between work–life balance and engagement.

Accordingly, we propose the following hypothesis:

*Hypothesis 2*: Authoritarian leadership moderates the negative relationship between illegitimate tasks and work–life balance, such that this relationship is stronger when authoritarian leadership is higher.

### 2.4 The conditional indirect association

The conditional indirect hypothesis combines the distinct predictions of the two theories without merging their explanatory roles. Work–family boundary theory explains why authoritarian leadership changes the strength of the initial association between illegitimate tasks and work–life balance. When authoritarian leadership is high, employees have less latitude to negotiate task ownership, completion timing, and priorities. Illegitimate tasks should therefore be more strongly associated with unfavorable work–life balance appraisals.

CAPS explains how this difference in work–life balance appraisal is associated with the remainder of the indirect process. Less favorable work–life balance is associated with lower work engagement, which is, in turn, associated with lower employee-reported work role performance. Consequently, the negative indirect association between illegitimate tasks and work role performance through work–life balance and work engagement should become stronger as authoritarian leadership increases.

**Figure 1:**
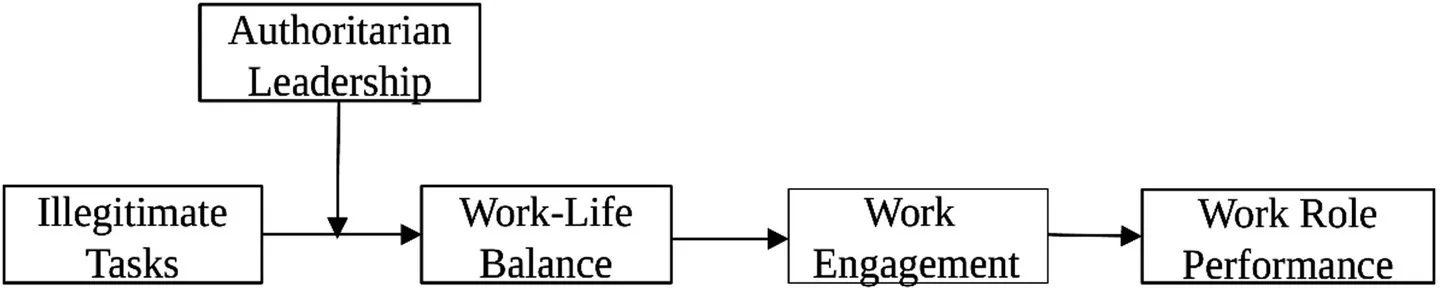
Research model. The figure presents the focal hypothesized relationships. Direct paths, ancillary paths, and control variables included in the empirical analyses are omitted for clarity.

This argument does not propose that authoritarian leadership moderates the later associations from work–life balance to work engagement or from work engagement to work role performance. Work–family boundary theory explains only the first-stage moderation, whereas CAPS explains how the resulting difference in cognitive appraisal is associated with affective–motivational investment and performance (Figure 1).

*Hypothesis 3*: Authoritarian leadership moderates the indirect association between illegitimate tasks and employee-reported work role performance through work–life balance and work engagement, such that this negative indirect association is stronger when authoritarian leadership is higher.

## 3 Materials and methods

### 3.1 Research setting, sample, and procedure

We collected three-wave, time-lagged survey data from engineering consulting professionals employed in 52 firms in Southwest China. Participating firms were recruited through an industry association and alumni networks. With organizational approval, human resource contacts invited employees whose primary responsibilities involved technical consulting work; administrative and logistics personnel were excluded. Data collection began in March 2025. At Time 1, 765 of the 860 invited employees completed the survey and reported demographic information, illegitimate tasks, and authoritarian leadership. Approximately 1 month later, participants completed the Time 2 survey assessing work–life balance and work engagement. One month after Time 2, they completed the Time 3 survey assessing work role performance. Responses across waves were matched using self-generated identification codes based on the last four digits of participants’ mobile phone numbers and the last two digits of their birth year. Participants were informed that their responses would remain confidential, would be used only for research purposes, and would be submitted directly to the research team. After matching responses across the three waves, we obtained 615 valid matched questionnaires. We screened the matched responses for data quality, including unusually short completion times, patterned responding, and missing values on the focal variables. No cases were excluded during this screening process. Therefore, the final sample consisted of 615 valid matched responses, representing a valid matched response rate of 71.5% relative to the initially distributed questionnaires and a three-wave retention rate of 80.4% relative to the Time 1 respondents.

The final sample consisted of 321 male employees (52.2%) and 294 female employees (47.8%). Regarding age, 19 participants (3.1%) were 20 years old or younger, 246 participants (40.0%) were between 21 and 30 years old, 205 participants (33.3%) were between 31 and 40 years old, 118 participants (19.2%) were between 41 and 50 years old, and 27 participants (4.4%) were 51 years old or older. Regarding educational attainment, 67 participants (10.9%) had a high school education or below, 232 participants (37.7%) had a junior college education, 249 participants (40.5%) had a bachelor’s degree, and 67 participants (10.9%) had a graduate degree or above.

### 3.2 Measures

We measured all focal constructs using established scales. Scales originally developed in English were translated into Chinese and independently back-translated following Brislin’s (1970) procedure. Differences between the original and back-translated versions were reviewed and resolved before the survey was administered. Unless otherwise indicated, items were rated on five-point response scales ranging from 1 (“strongly disagree”) to 5 (“strongly agree”).

#### 3.2.1 Illegitimate tasks

We measured illegitimate tasks using the scale developed by Semmer et al. (2010). The measure distinguishes between unreasonable tasks, which employees perceive as falling outside their legitimate role responsibilities, and unnecessary tasks, which employees perceive as avoidable or lacking a clear purpose. A sample item from the unreasonable-task subscale was, “Do you have to carry out tasks which you believe should be done by someone else?” The measure includes two related dimensions: unreasonable tasks and unnecessary tasks. Consistent with our construct-level hypotheses, we combined the items from the two dimensions to form an overall illegitimate-tasks score. Cronbach’s alpha for the overall scale was 0.873.

#### 3.2.2 Authoritarian leadership

We measured authoritarian leadership using the scale developed by Cheng et al. (2004). A sample item is “My supervisor asks me to obey his/her instructions completely.” In this study, Cronbach’s alpha for this scale was 0.946.

#### 3.2.3 Work–life balance

We measured work–life balance using the scale developed by Brough et al. (2014). The scale consists of four items and assesses employees’ subjective evaluation of the compatibility between work and nonwork activities. A sample item is “Overall, I believe that my work and non-work life are balanced.” In this study, Cronbach’s alpha for this scale was 0.882.

#### 3.2.4 Work engagement

We measured work engagement using the short version of the Utrecht Work Engagement Scale (UWES-9) developed by Schaufeli et al. (2006). The scale includes three dimensions: vigor, dedication, and absorption. A sample item is “At my work, I feel bursting with energy.” In this study, Cronbach’s alpha for this scale was 0.944.

#### 3.2.5 Work role performance

We measured work role performance using the scale developed by Griffin et al. (2007). This scale was designed to capture work behaviors in uncertain environments and includes three dimensions: individual task proficiency, individual task adaptivity, and individual task proactivity, with nine items in total. A sample item is “Carried out the core parts of your job well.” In this study, Cronbach’s alpha for this scale was 0.945.

### 3.3 Analytical strategy

We first assessed the measurement model using confirmatory factor analysis in AMOS 26.0. Model fit was evaluated using χ^2^, CFI, TLI, RMSEA, and SRMR. The proposed five-factor model treated illegitimate tasks, authoritarian leadership, work–life balance, work engagement, and work role performance as distinct latent constructs and was compared with alternative models that combined theoretically distinct constructs. The single-factor model was included as an alternative measurement model rather than as a test of common method variance. We assessed common method variance by comparing the five-factor model with an unmeasured latent method construct model. Gender, age, and educational level were included as controls. Because age and organizational tenure were highly correlated (*r* = 0.896), we excluded organizational tenure to avoid redundancy.

We tested the hypotheses in Mplus 8.3 using the overall illegitimate-tasks score. We estimated the theoretically ordered indirect association from illegitimate tasks to work role performance through work–life balance and work engagement, the interaction between illegitimate tasks and authoritarian leadership in predicting work–life balance, and the corresponding conditional serial indirect associations. Simple slopes and conditional indirect effects were examined at one standard deviation below the mean, at the mean, and one standard deviation above the mean of authoritarian leadership. Indirect effects and the index of moderated serial mediation were evaluated using 5,000 bias-corrected bootstrap samples and 95% confidence intervals. We report unstandardized coefficients, standard errors, and confidence intervals. Because work–life balance and work engagement were measured concurrently, their ordering was theoretically specified rather than temporally established; thus, the findings are interpreted as theoretically ordered associations rather than evidence of temporal or causal sequence.

## 4 Results

### 4.1 Measurement model and assessment of common method variance

The proposed five-factor model, which specified illegitimate tasks, authoritarian leadership, work–life balance, work engagement, and work role performance as distinct constructs, provided the best fit among the models examined, *χ*^2^(692) = 2,518.901, *χ*^2^/df = 3.640, CFI = 0.903, TLI = 0.897, RMSEA = 0.066, and SRMR = 0.048. Models that combined additional constructs showed progressively poorer fit. These comparisons support the discriminant validity of the five focal constructs (Table 1).

**Table 1:** Results of confirmatory factor analyses.

Model	χ^2^	df	χ^2^/df	CFI	TLI	RMSEA	SRMR
IT, AL, WLB, WE, RP	2518.901	692	3.640	0.903	0.897	0.066	0.048
IT+AL, WLB, WE, RP	3939.429	696	5.660	0.829	0.818	0.087	0.103
IT+AL, WLB + WE, RP	4914.349	699	7.031	0.777	0.764	0.099	0.116
IT+AL + WLB + WE, RP	6769.098	701	9.656	0.679	0.661	0.119	0.095
IT+AL + WLB + WE+RP	9070.525	702	12.921	0.558	0.533	0.139	0.111

*N* = 615. IT, illegitimate tasks; AL, authoritarian leadership; WLB, work–life balance; WE, work engagement; RP, work role performance. Commas indicate constructs modeled as distinct factors; plus signs indicate constructs combined into one factor.

Because all focal variables were employee-reported, we considered the potential influence of common method variance. Temporal separation across the three survey waves was intended to reduce, although not eliminate, concerns associated with common-source measurement (Fuller et al., 2016). We further assessed common method variance by comparing the five-factor measurement model with an unmeasured latent method construct model. Adding the method factor improved model fit: CFI increased from 0.903 to 0.950, TLI increased from 0.897 to 0.943, RMSEA decreased from 0.066 to 0.049, and SRMR decreased from 0.048 to 0.025. Although the fit indices improved after the inclusion of the method factor, the fit of the original five-factor model remained largely within commonly accepted ranges, suggesting that common method variance was unlikely to pose a serious threat to the validity of the findings. Nevertheless, these results indicate that the method factor captured some shared variance. Accordingly, common method variance cannot be entirely ruled out and should be considered when interpreting the findings.

### 4.2 Descriptive statistics and correlations

Table 2 reports the descriptive statistics, reliabilities, and zero-order correlations. Illegitimate tasks were positively associated with authoritarian leadership (*r* = 0.473, *p* < 0.01) and negatively associated with work–life balance (*r* = −0.543), work engagement (*r* = −0.628), and employee-reported work role performance (*r* = −0.581), all *p*s < 0.01. Work–life balance was positively associated with work engagement (*r* = 0.511) and work role performance (*r* = 0.515), and work engagement was positively associated with work role performance (*r* = 0.535), all *p*s < 0.01. Authoritarian leadership was negatively associated with work–life balance (*r* = −0.442), work engagement (*r* = −0.554), and work role performance (*r* = −0.482), all *p*s < 0 0.01. These correlations were consistent with the proposed pattern of relationships, although the hypotheses were evaluated using the multivariate models reported below.

**Table 2:** Means, standard deviations, reliabilities, and correlations.

Variable	Gender	Age	Edu	AL	IT	WLB	WE	RP
Gender	–							
Age	−0.005	–						
Edu	−0.071	−0.385^**^	–					
AL	0.072	−0.001	−0.051	(0.946)				
IT	0.166^**^	−0.006	−0.119^**^	0.473^**^	(0.873)			
WLB	−0.114^**^	0.058	0.036	−0.442^**^	−0.543^**^	(0.882)		
WE	−0.180^**^	−0.017	0.088^*^	−0.554^**^	−0.628^**^	0.511^**^	(0.944)	
RP	−0.070	0.007	0.069	−0.482^**^	−0.581^**^	0.515^**^	0.535^**^	(0.945)
M	1.480	2.820	2.510	2.355	2.589	3.582	3.429	3.460
SD	0.500	0.927	0.829	1.189	1.034	1.136	1.178	1.139

*N* = 615. Cronbach’s alpha coefficients are reported in parentheses on the diagonal. IT, illegitimate tasks; AL, authoritarian leadership; WLB, work–life balance; WE, work engagement; RP, work role performance. ∗*p* < 0.05; ∗∗*p* < 0.01, two-tailed.

### 4.3 Hypothesis tests

To test Hypothesis 1, we estimated the theoretically ordered indirect model in Mplus 8.3 using 5,000 bias-corrected bootstrap samples. Controlling for gender, age, education, and authoritarian leadership, illegitimate tasks were negatively associated with work–life balance (*b* = −0.315, SE = 0.047, *p* < 0.001). Work–life balance was positively associated with work engagement (*b* = 0.240, SE = 0.043, *p* < 0.001), and work engagement was positively associated with employee-reported work role performance (*b* = 0.214, SE = 0.051, *p* < 0.001).

The serial indirect association between illegitimate tasks and work role performance through work–life balance and work engagement was negative and statistically different from zero (indirect effect = −0.016, 95% bias-corrected bootstrap CI [−0.032, −0.007]). The indirect association between illegitimate tasks and work engagement through work–life balance accounted for approximately 23.7% of the total association between illegitimate tasks and work engagement. This proportion is reported descriptively and should not be interpreted as evidence of causal mediation. Thus, Hypothesis 1 was supported.

The specific indirect associations through work–life balance alone (indirect effect = −0.073, 95% bias-corrected bootstrap CI [−0.120, −0.040]) and through work engagement alone (indirect effect = −0.052, 95% bias-corrected bootstrap CI [−0.088, −0.026]) were also statistically different from zero. The direct association between illegitimate tasks and work role performance remained significant (*b* = −0.220, SE = 0.041, *p* < 0.001), indicating that the indirect pathways did not account for the entire association. Because work–life balance and work engagement were assessed during the same survey wave, these results should be interpreted as consistent with the proposed theoretical ordering rather than as establishing temporal precedence between the two mediators.

As shown in Table 3, the interaction between illegitimate tasks and authoritarian leadership was negatively associated with work–life balance (*b* = −0.088, SE = 0.034, *p* = 0.009, 95% bias-corrected bootstrap CI [−0.153, −0.022]). Simple-slope analyses showed that illegitimate tasks were negatively associated with work–life balance when authoritarian leadership was low (*b* = −0.229, SE = 0.067, 95% CI [−0.361, −0.096]), at its mean (*b* = −0.317, SE = 0.047, 95% CI [−0.409, −0.227]), and high (*b* = −0.406, SE = 0.046, 95% CI [−0.493, −0.312]). The difference between the slopes at high and low authoritarian leadership was −0.177 (SE = 0.068, 95% CI [−0.307, −0.044]). Thus, the negative association between illegitimate tasks and work–life balance became stronger as authoritarian leadership increased, supporting Hypothesis 2.

**Table 3:** Moderating effect of authoritarian leadership and simple-slope results.

Effect	b	SE	95% BC bootstrap CI
IT × AL → WLB	−0.088	0.034	[−0.153, −0.022]
IT → WLB at Low AL	−0.229	0.067	[−0.361, −0.096]
IT → WLB at Mean AL	−0.317	0.047	[−0.409, −0.227]
IT → WLB at High AL	−0.406	0.046	[−0.493, −0.312]
High AL vs. Mean AL	−0.088	0.034	[−0.153, −0.022]
Mean AL vs. Low AL	−0.088	0.034	[−0.153, −0.022]
High AL vs. Low AL	−0.177	0.068	[−0.307, −0.044]

*N* = 615. IT, illegitimate tasks; AL, authoritarian leadership; WLB, work–life balance. Unstandardized coefficients are reported. Simple slopes were estimated at one standard deviation below the mean, at the mean, and one standard deviation above the mean of authoritarian leadership.

**Figure 2:**
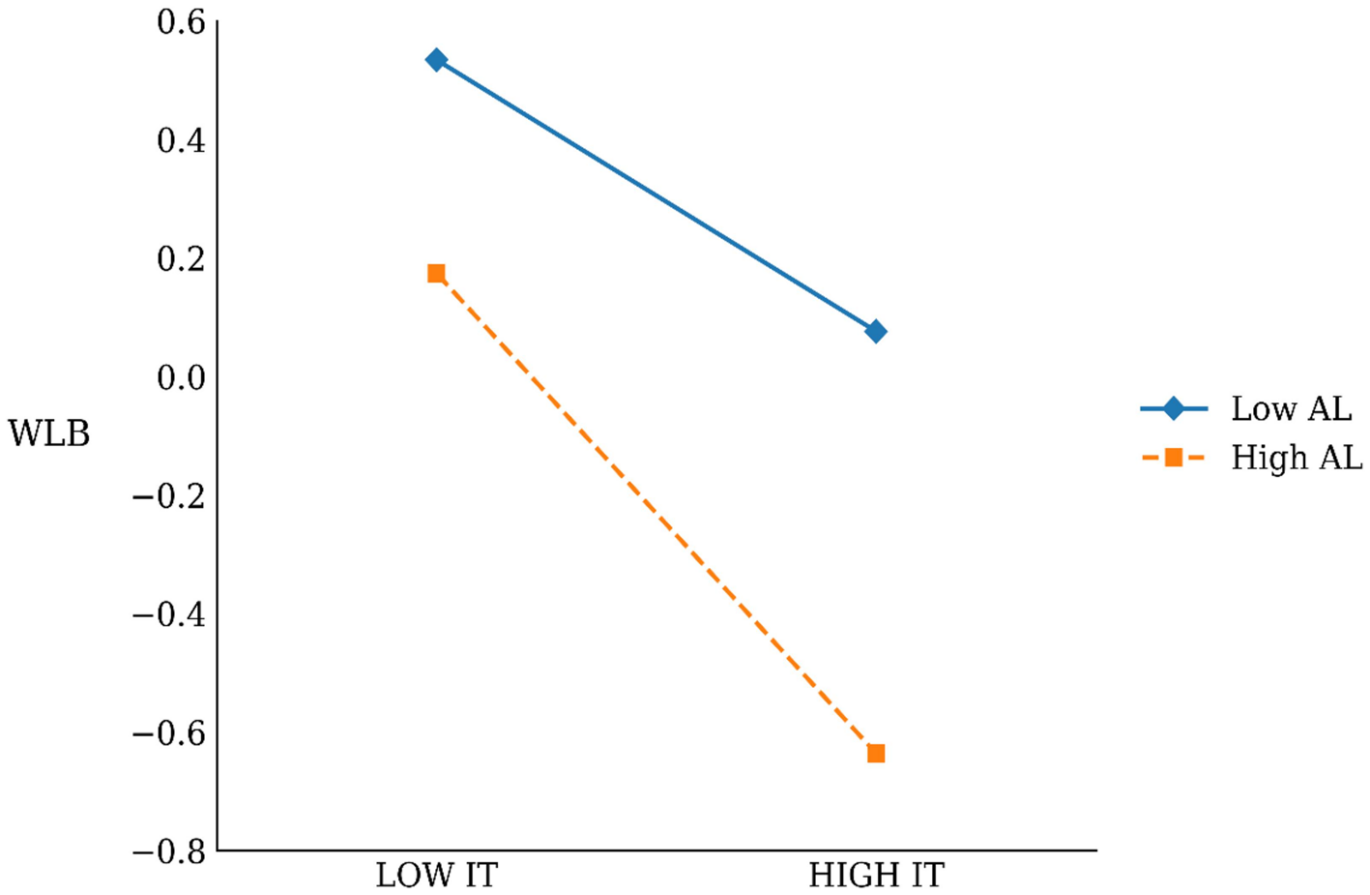
Interaction between illegitimate tasks and authoritarian leadership in predicting work–life balance. The simple slope was *b* = −0.229 at low authoritarian leadership and *b* = −0.406 at high authoritarian leadership.

To facilitate interpretation of the moderating effect, we plotted the simple slopes, as shown in Figure 2.

Tables 4, 5 show that the conditional serial indirect association between illegitimate tasks and work role performance through work–life balance and work engagement was −0.012 at low authoritarian leadership, −0.016 at the mean, and −0.021 at high authoritarian leadership. The corresponding 95% bias-corrected bootstrap confidence intervals were [−0.027, −0.003], [−0.032, −0.007], and [−0.040, −0.009], respectively. Thus, the negative indirect association became stronger as authoritarian leadership increased. The difference between the indirect associations at high and low authoritarian leadership was −0.009, with a 95% bias-corrected bootstrap confidence interval of [−0.020, −0.003]. The index of moderated serial mediation was also negative and statistically different from zero (index = −0.005, 95% CI [−0.010, −0.002]). Together, these results indicate that authoritarian leadership strengthened the negative indirect association between illegitimate tasks and work role performance through work–life balance and work engagement, supporting Hypothesis 3.

**Table 4:** Conditional serial indirect associations.

AL level	Conditional indirect effect	SE	95% BC bootstrap CI
Low AL (−1 SD)	−0.012	0.006	[−0.027, −0.003]
Mean AL	−0.016	0.007	[−0.032, −0.007]
High AL (+1 SD)	−0.021	0.008	[−0.040, −0.009]

**Table 5:** Differences in conditional serial indirect associations.

Comparison	Difference	SE	95% BC bootstrap CI
High AL vs. Mean AL	−0.005	0.002	[−0.010, −0.002]
Mean AL vs. Low AL	−0.005	0.002	[−0.010, −0.002]
High AL vs. Low AL	−0.009	0.004	[−0.020, −0.003]
Index of Moderated Serial Mediation	−0.005	0.002	[−0.010, −0.002]

## 5 Discussion

The findings support the CAPS-based account of the proposed indirect process. Illegitimate tasks operated as situational stimuli associated with less favorable work–life balance appraisals. Lower work–life balance was associated with lower work engagement, representing the affective–motivational unit in the model, and lower work engagement was, in turn, associated with lower employee-reported work role performance. The significant serial indirect association is therefore consistent with the theoretically ordered CAPS-based stimulus–appraisal–motivation–performance sequence. However, because work–life balance and work engagement were measured concurrently, the findings support a theoretically ordered indirect association rather than a definitive temporal or causal process.

Separately, the findings support the work–family boundary explanation of the moderating role of authoritarian leadership. The negative association between illegitimate tasks and work–life balance became stronger as authoritarian leadership increased. From a work–family boundary perspective, authoritarian leaders act as work-domain boundary managers who restrict employees’ latitude to negotiate task ownership, completion timing, and priorities. This restricted latitude makes illegitimate tasks more difficult to accommodate within employees’ work and nonwork arrangements, thereby strengthening the initial negative association between illegitimate tasks and work–life balance. The theory is used to explain this first-stage moderation rather than the subsequent mediation process.

### 5.1 Theoretical contributions

The findings make three contributions by positioning CAPS as the primary theory of the indirect process, identifying work–life balance as one complementary cross-domain pathway, and using work–family boundary theory to explain the moderating role of authoritarian leadership.

First, this study clarifies CAPS as the primary process theory explaining how illegitimate tasks are associated with employee-reported work role performance through a theoretically ordered stimulus–appraisal–motivation–performance sequence. CAPS conceptualizes illegitimate tasks as situational stimuli that activate work–life balance appraisals; these appraisals are associated with work engagement as an affective–motivational unit and, in turn, work role performance (Mischel and Shoda, 1995; Shoda and Mischel, 2000). Work–family boundary theory serves a distinct and narrower role by explaining why authoritarian leaders, as work-domain boundary managers, strengthen the initial association between illegitimate tasks and work–life balance by constraining employees’ latitude to negotiate task ownership, completion timing, and priorities (Ashforth et al., 2000; Clark, 2000). This theoretical division assigns each theory a clear explanatory role: CAPS accounts for the indirect process, whereas work–family boundary theory accounts for its first-stage boundary condition.

Second, by applying CAPS to the full stimulus–appraisal–motivation–performance sequence, the study identifies work–life balance as one cross-domain appraisal pathway through which illegitimate tasks may be associated with engagement and performance. Prior research has emphasized affective reactions, perceived unfairness, threats to self-worth, and occupational identity as explanations for the consequences of illegitimate tasks (Ahmed et al., 2018; Ding and Kuvaas, 2023; Eatough et al., 2016). The present findings do not establish work–life balance as the sole or primary mechanism. Rather, they show that employees’ appraisal of work–nonwork compatibility represents an additional pathway that complements these established explanations. Moreover, work–life balance accounted for approximately 23.7% of the total association between illegitimate tasks and work engagement, indicating that a substantial part of this association remained independent of the proposed appraisal pathway.

Third, this study clarifies the role of authoritarian leadership as a contextual condition that shapes employees’ appraisal of illegitimate tasks. Existing studies often treat leadership style as a general managerial context associated with employee attitudes or performance (Judge and Piccolo, 2004; Pizzolitto et al., 2023). The present findings indicate that authoritarian leadership is particularly relevant at the initial appraisal stage. When authoritarian leadership is low, employees may have greater latitude to clarify responsibilities, negotiate task timing, or reprioritize assigned work. When it is high, these options are more restricted, making illegitimate tasks more strongly associated with unfavorable work–life balance appraisals. This contribution extends work–family boundary theory by identifying authoritarian leaders as work-domain boundary managers whose control over task ownership, completion timing, and priorities shapes the negotiability of illegitimate work demands.

### 5.2 Practical implications

First, managers should shift from trying to eliminate all miscellaneous work to improving how the rationale for such work is communicated. In project-based settings, it is unrealistic to remove all low-value administrative or coordination tasks. However, organizations should not rely solely on professionals’ individual adjustment. Instead, they should reduce unnecessary disruptions to employees’ work and nonwork arrangements by structurally separating technical and administrative functions where possible. For example, organizations may establish shared administrative support centers to handle routine reporting, documentation, and coordination tasks, thereby reducing the extent to which such demands interfere with professionals’ core work.

Because the harm of illegitimate tasks depends partly on whether employees interpret such tasks as unnecessary or unreasonable, managers should clearly explain the connection between non-core tasks and project delivery, compliance requirements, or client needs. For instance, when complex procedures or reporting requirements are unavoidable, managers should explain how these activities contribute to overall project delivery or client compliance. Such explanations can reduce the likelihood that employees interpret these tasks as lacking necessity or falling outside their role responsibilities. This form of cognitive-level intervention can help reduce task disturbances that employees may otherwise appraise as unnecessary or unreasonable (Ding and Kuvaas, 2023; Semmer et al., 2010, 2015).

Second, organizations should be attentive to the amplifying effect of authoritarian management when illegitimate tasks are assigned and should establish task-negotiation and scheduling mechanisms that combine flexibility with empowerment. The findings suggest that authoritarian leadership strengthens the negative association between illegitimate tasks and work–life balance. Thus, when managing knowledge workers, leaders should avoid relying excessively on directive practices that require immediate and unquestioned obedience. When professionals must handle non-core tasks, managers should provide discretion over the timing and manner of task completion. For example, employees could be allowed to complete such tasks during intervals between project milestones rather than having their core work interrupted immediately. Such flexibility can buffer the pressure associated with illegitimate tasks and provide employees with the psychological space needed to improve their ability to coordinate work and nonwork activities and restore work–life balance (Ashforth et al., 2000; Karasek, 1979).

Third, organizations should protect deep work at the level of work design. Because unnecessary tasks, such as frequent internal reporting and lengthy milestone approvals, can interrupt cognitive continuity, organizations should protect periods of uninterrupted core work through institutionalized arrangements. For example, they may introduce no-meeting days, protected focus periods, or rules limiting non-urgent administrative requests during technically intensive work periods. By temporally and procedurally separating core technical work from administrative trivialities, organizations can reduce the extent to which low-value tasks interfere with professionals’ attention, cognitive continuity, and work engagement (Baethge et al., 2016; Christian et al., 2011; Leroy, 2009).

Finally, organizations should use compensatory support when illegitimate tasks cannot be avoided. When professionals must complete tasks that are low in value or weakly aligned with their role responsibilities, managers can combine task explanations with subsequent opportunities for high-autonomy and high-value core work. They can also provide emotional support by acknowledging the disruption caused by such tasks and helping employees restore a more manageable work–life arrangement. These practices may help reduce the likelihood that unfavorable work–life balance appraisals are associated with lower engagement and lower employee-reported work role performance (Christian et al., 2011; Ding and Kuvaas, 2023; Semmer et al., 2010).

### 5.3 Limitations and future research

Although this study adopted a three-wave time-lagged design, several limitations should be noted. First, all core variables were based on employee self-reports. Although the three-wave design helped reduce the risk of common method bias, single-source data may still inflate the observed relationships among variables. Future research could adopt multi-source designs, such as having employees report illegitimate tasks while supervisors rate work role performance. Researchers could also combine survey responses with behavioral or archival data to further strengthen the robustness of the findings. The improved fit of the latent method model also indicates that shared method variance may have influenced the estimated relationships. The observed associations should therefore be interpreted cautiously.

Second, work–life balance and work engagement were assessed during the same survey wave. Although their ordering was theoretically derived from their distinct roles as cognitive appraisal and affective–motivational investment, the design does not establish that work–life balance temporally preceded work engagement. Nor did the study compare the proposed ordering with reverse or parallel mediation models. Future research should measure the two mediators at separate time points and compare theoretically plausible alternative specifications. Accordingly, the present findings should be interpreted as evidence of a theoretically ordered indirect association rather than a definitive causal sequence.

Third, this study was unable to capture employees’ immediate psychological reactions when illegitimate tasks occurred. The current design examined relationships among variables across time, but it did not fully capture the momentary psychological changes triggered by specific illegitimate tasks. Because illegitimate tasks are often sudden and context-dependent, future research could use experience sampling or diary methods to examine employees’ real-time emotional fluctuations and cognitive appraisal processes when they encounter unreasonable or unnecessary assignments. Such designs would provide a more fine-grained understanding of how illegitimate tasks activate psychological states in daily work.

Finally, the generalizability of the findings across cultural contexts requires further examination. The sample was drawn primarily from organizations operating in an Eastern, high power-distance context, where authoritarian leadership may be more prevalent and more normatively accepted. Future research could conduct cross-cultural comparisons to examine whether illegitimate tasks produce similar effects in Western contexts characterized by flatter management structures, or whether cultural differences in responsibility boundaries and authority relations shape distinct patterns of harm. Such research would help clarify the boundary conditions of the model and its applicability across cultural settings.

## 6 Conclusion

This study used CAPS as the primary theoretical framework to examine how illegitimate tasks are associated with employee-reported work role performance among engineering consulting professionals. CAPS conceptualizes illegitimate tasks as situational stimuli that activate work–life balance appraisals, which are associated with work engagement and, in turn, work role performance. Work–family boundary theory explains why authoritarian leadership strengthens the initial association: authoritarian leaders act as work-domain boundary managers who restrict employees’ latitude to negotiate task ownership, completion timing, and priorities. The findings supported the proposed serial indirect association and showed that it became more negative as authoritarian leadership increased. By assigning distinct roles to the two theories, the study identifies a CAPS-based stimulus–appraisal–motivation–performance process and a boundary-theory explanation for when its initial association is stronger. Because work–life balance and work engagement were measured concurrently, these findings should be interpreted as supporting a theoretically ordered indirect association rather than establishing a definitive temporal or causal sequence.

## Data Availability

The datasets presented in this article are not readily available because the raw questionnaire data involves the privacy of enterprise employees and internal business information. For the protection of participants’ personal privacy and confidential organizational data, the original dataset will not be shared with third parties. Requests to access the datasets should be directed to yangtao@gxlvtc.edu.cn.
